# Supplementary material for: Newborn Screening for Metachromatic Leukodystrophy: A Systematic Literature Review
Source: Int J Neonatal Screen. 2025 Nov 5;11(4):103. doi: 10.3390/ijns11040103 (PMC12641716; doi:10.3390/ijns11040103)
Supplement: Supplementary file 1 [file IJNS-11-00103-s001.zip › IJNS-3805241-supplementary.pdf]

Supplementary Materials

Table S1. Searches for 'newborn screening' (searches performed on 19 February 2025).

		Embase	MEDLINE	Cochrane
1	exp mass spectrometry/ or exp tandem mass spectrometry/ or exp newborn screening/ or ((new born or new-born or newborn) adj3 screening).mp.	712564	314716	2887
2	exp metachromatic leukodystrophy/ or (metachromatic leukodystrophy or MLD or Arylsulfatase A Deficiency).tw.	7626	4790	511
3	1 and 2	203	83	1
4	limit 3 to yr="2015 -Current"	151	47	1
5	limit 4 to (animals and animal studies)	10	7	1
6	4 not 5	141	40	0
**Total**		**181**		

Table S2. Searches for ‘treatment’ (searches performed on 19 February 2025).

		Embase	MEDLINE	Cochrane
1	(atidarsagene autotemcel or arsa-cel or libmeldy or lenmeldy or (hematopoietic adj3 transplantation) or HSCT).tw.	83331	43019	4457
2	exp metachromatic leukodystrophy/ or (metachromatic leukodystrophy or MLD or Arylsulfatase A Deficiency).tw.	7626	4790	511
3	1 and 2	293	106	3
4	limit 3 to yr="2015 -Current"	214	70	2
5	limit 4 to (animals and animal studies)	5	6	2
6	4 not 5	209	64	0
**Total**			**273**	

Table S3. Inclusion criteria.

Category	Inclusion criteria
Patients	Newborns undergoing NBS for MLD
Interventions	Not restricted by intervention
Comparator	Not restricted by comparators
Outcomes	*NBS outcomes*<br>• Sensitivity, specificity, and predictive value of MLD screening tests<br>• Feasibility, scalability, and cost-effectiveness of NBS programs<br>• Ethical, legal, and social considerations<br>*Availability of screening tests*<br>• Types of screening assays<br>• Validation, reliability, and implementation in national programs<br>*Diagnostic confirmation methods*<br>• Algorithms for confirmatory diagnosis<br>• False-positive and false-negative rates<br>• Turnaround time for confirmation<br>*Identification through population-based screening*<br>• Number and characteristics of infants identified through NBS<br>• Follow-up and monitoring strategies<br>*Impact of early identification on health outcomes*<br>• Time to diagnosis and treatment initiation<br>• Disease progression, neurological and functional outcomes<br>• Survival, HRQoL, and treatment effectiveness
Study design	Not restricted by study design
Language	English
Publication type	Not restricted by publication type
Countries	Not restricted by country
Date restrictions	Studies published from 2015 to present

HRQoL, health-related quality of life; MLD, metachromatic leukodystrophy; NBS, newborn screening.
